# Somatic mutations in cancer development

**DOI:** 10.1186/1476-069X-10-S1-S12

**Published:** 2011-04-05

**Authors:** Lucio Luzzatto

**Affiliations:** 1 Scientific Director, Istituto Toscano Tumori, Florence, Italy

## Abstract

The transformation of a normal cell into a cancer cell takes place through a sequence of a small number of discrete genetic events, somatic mutations: thus, cancer can be regarded properly as a genetic disease of somatic cells. The analogy between evolution of organisms and evolution of cell populations is compelling: in both cases what drives change is mutation, but it is Darwinian selection that enables clones that have a growth advantage to expand, thus providing a larger target size for the next mutation to hit. The search for molecular lesions in tumors has taken on a new dimension thanks to two powerful technologies: the micro-arrays for quantitative analysis of global gene expresssion (the *transcriptome*); and ‘deep’ sequencing for the global analysis of the entire genome (or at least the *exome*). The former offers the most complete phenotypic characterization of a tumor we could ever hope for – we could call this the *ultimate phenotype*; the latter can identify all the somatic mutations in an individual tumor – we could call this the *somatic genotype*. However, there is definitely the risk that while we are ‘drowned by data, we remain thirsty for knowledge’. If we want to heed the teachings of Lorenzo Tomatis, I think the message is clear: we ought to take advantage of the new powerful technologies – not by becoming their slaves, but remaining their masters. Identifying somatic mutations in a tumor is important not because it qualifies for ‘oncogenomics’, but because through a deeper understanding of the nature of that particular tumor it can help us to optimize therapy or to design new therapeutic approaches.

## Introduction

**Figure 1:**
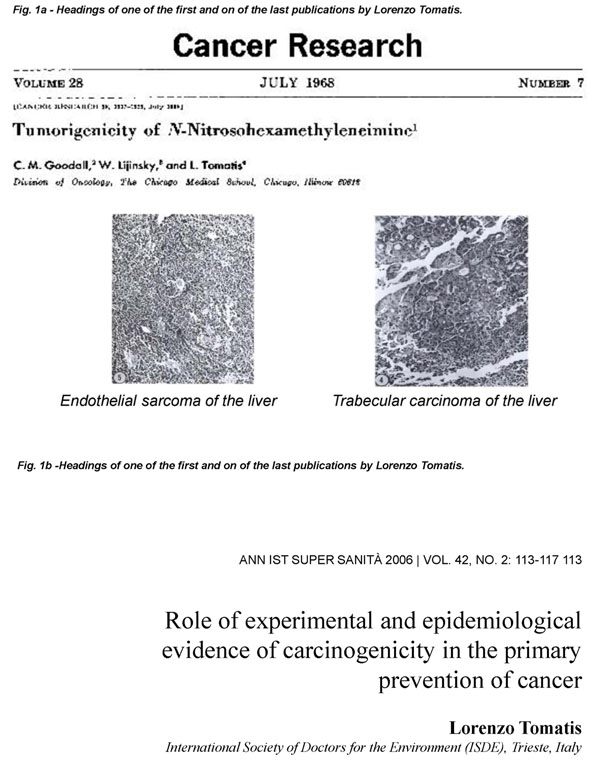
Headings of one of the first and on of the last publications by Lorenzo Tomatis.

Lorenzo Tomatis was a towering figure in the study of cancer and cancer epidemiology: not just because from 1982 to 1993 he was the Director of the *International Agency for Research against Cancer* (IARC), but even more because he commanded immense international respect as a scientist ahead of his time in the understanding of the environmental causes of cancer. Tomatis’ major influence in this area spanned some four decades[1,2] (see Figs. 1a and 1b). I never worked with Renzo, but I have vivid memories of many encounters and discussions I had with him, both about science and about research policies: and I am forever grateful for what I learnt from him.

**Figure 2:**
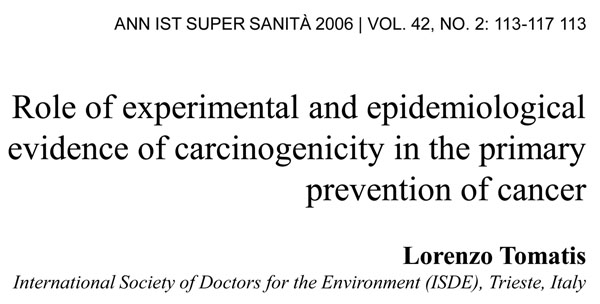
**Pedigree of a family with a high rate of breast cancer and ovarian cancer:** the increased tendency to developing cancer shows a Mendelian autosomal dominant pattern of inheritance, suggesting that a single gene is largely responsible.

**Figure 3:**
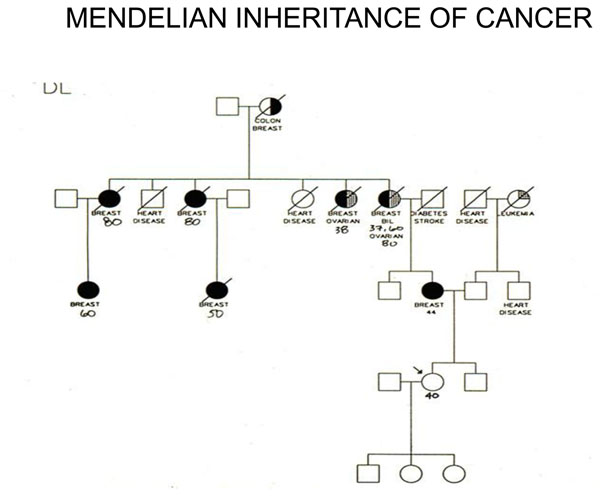
**In this extended family there were 3 cases of hairy cell leukaemia (HCL): their co-existence can be hardly a coincidence, since HCL is one of the rarest** forms of B cell leukaemia. Here the pattern is not Mendelian, suggesting that several genes and/or environmental factors are involved.

**Figure 4:**
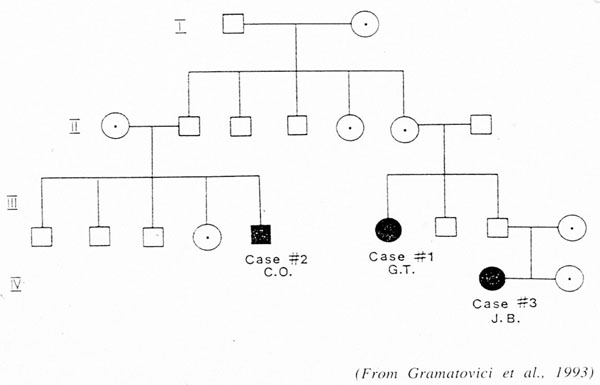
Three patients with hairy cell leukaemia in the same family.

**Figure 5:**
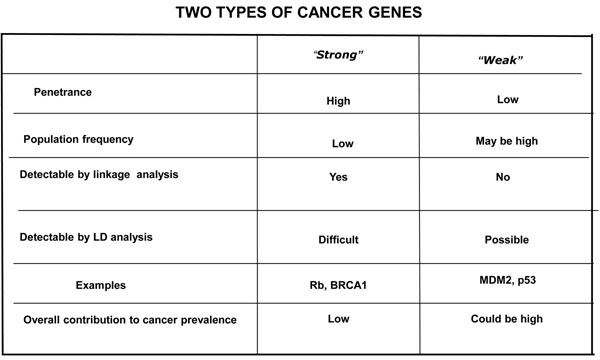
Meta-analysis of the quantitative effect of a polymorphic allele of the TGF b receptor gene on the frequency of some types of tumors.

**Figure 6:**
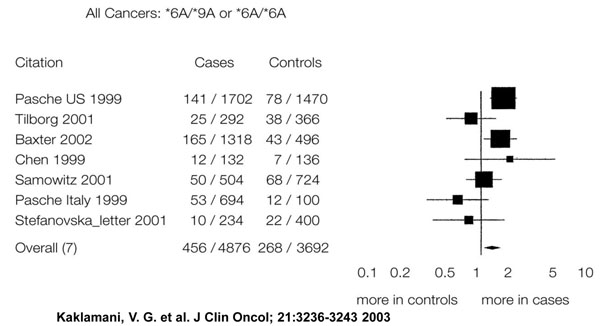
A genetic polymorphism in the coding sequence of the TGF-β receptor gene influences the risk of cancer.

My job today is to discuss the role of somatic mutations in oncogenesis. In a nutshell, and using a time-honoured terminology of medicine, if heredity and environment are the aetiology of cancer, somatic mutations are the essence of its pathogenesis. With respect to heredity, it is abundantly clear that one never does inherit cancer, but rather one may inherit an increased risk of cancer[3]. In first approximation, some mutant genes entail a very high risk of cancer, so much so that they behave as Mendelian dominants (see Fig. 2), and they are therefore called high penetrance (cancer susceptibility) genes. These include (i) tumor suppressors (*e.g. p53*, *APC*, *GPC3*, *VHL*, *CDKN2A*, *MEN1*), (ii) oncogenes (*e.g. PDGFRA*, *KIT*, *MET*, *RET*), and (iii) genes required for genome stability (*e.g. ATM*, *BLM*, *FANCA*, *BRCA2*, *MSH2*, *XPA*). Known high penetrance genes number by now several dozens[4]: they may have tumor (site) specificity (*e.g. BRCA2*) or they may not (*e.g. p53*). In addition, numerous clinical observations (see for instance Fig. 3[5]) indicate that cancer susceptibility may ‘run in families’ in a more subtle way, and this has led to the notion of low penetrance (cancer susceptibility) genes[6]. These are important because they may contribute significantly to the cancer burden in a population (see Figure 4). Until recently, candidate low penetrance genes have been chased through (i) tests on first degree relatives[7], (ii) kinship analysis[8], (iii) studies on twins[9], and (iv) linkage disequilibrium analysis in appropriate populations[10-12] It must be admitted that until recently the yield has been limited, although individual examples have turned up, for instance among genes involved in signal transduction pathways (*e.g.* the TGFβ receptor: see Fig. 5), and numerous genes involved in DNA repair (Figure 6). Over the past 4 years, however, genome-wide association studies (GWAS) have become very popular: this is not a conceptually new approach, as it is merely an updated version of (iv), but it is made much more powerful through the availability of some millions single nucleotide polymorphisms (SNPs). Thanks to this increased power, many low penetrance genes or loci have been now identified, that affect the risk of individual types (or several types) of cancer – mostly by less than +/- 30% – in one or another population (see 40 references in webappendix of recent paper by Hartman et al.[13] .

With respect to the environment, I think the most lasting monumental memorial to Lorenzo is the series of IARC publications on carcinogenic agents which, in the jargon of the *cognoscentes*, are known simply as *The Monographs*. Rarely has an international agency been able to generate publications (each one the product of a collegial effort) with so much scientific content; even more rarely has this taken place consistently in dozens of volumes over some thirty years, to the extent that the Monographs are universally regarded as the ultimate authority on their individual topics; and probably never has a single person – namely Tomatis himself – through his scientific rigor, his incredible dedication, and his unique ability to catalyze consensus whenever possible, contributed so much to a successful venture of this nature.

**Figure 7:**
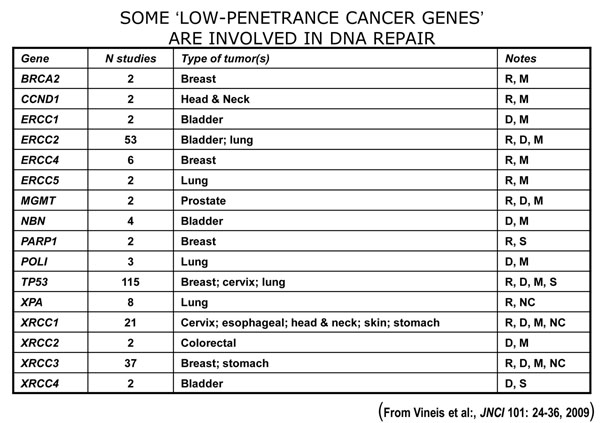
**A cartoon illustrating current views of the origin of cancer**, which is consequent on *n* successive somatic mutations. The final result is a clonal population of cells with highly disregulated growth. It can be presumed that in fact each one of the mutational steps entails a growth advantage, even if small: this increases the number of cells that can be targeted by the next mutation. The term *n-1* is used to indicate the penultimate step in the pathway, because the number n is not fixed: it is estimated that it may range, for the majority of tumors, from 3 to 6 or even more.

**Figure 8:**
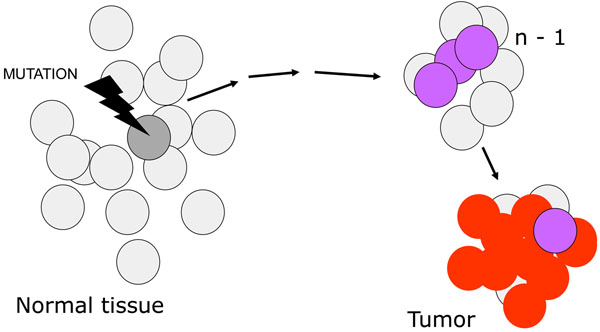


**Figure 9:**
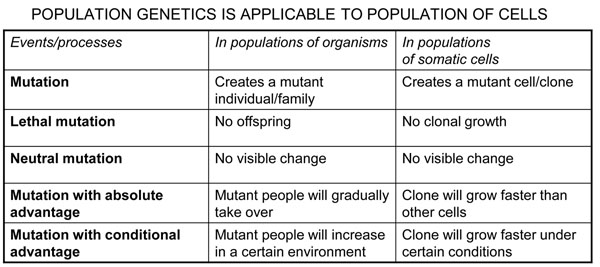
**Inherited mutations can increase cancer proneness through different mechanisms**. The top section of the cartoon is a schematic of the process outlined in Fig. 5. The middle section illustrates how an increased rate of somatic mutations can produce an accelerated rate of the oncogenic pathway: this is the case for instance for patients with Fanconi anemia, who have a serious defect in DNA repair and often develop cancer at a young age. The bottom section illustrate that the number of steps for a normal cell to become a cancer cell is cut by one if the first mutation is an inherited (germ-line) mutation rather than an acquired somatic mutation: this is the case for instance for patients who have an APC mutation and present with familial adenomatous polyposis.

The model of oncogenesis pioneered by John Cairns[14] contained already the key for reconciling aetiology and pathogenesis. The transformation of a normal cell into a cancer cell takes place through a sequence of a small number of discrete genetic events, somatic mutations (Figure 7): thus, cancer can be regarded properly as a genetic disease of somatic cells[3,15]. The analogy between evolution of organisms and evolution of cell populations is compelling (Figure 8): in both cases what drives change is mutation, but it is Darwinian selection that enables clones that have a growth advantage to expand, thus providing a larger target size for the next mutation to hit[14,16,17] (Figure 7). This model offers a simple interpretation to the mechanism of action of the aetiological factors we have mentioned. An environmental agent can increase the rate of somatic mutation (*i.e.*, it may be mutagenic, like ionizing radiation), or it can increase the rate of cell proliferation (as when *Helicobacter pylori* causes gastritis), or it may do both things (this is probably the case with the hepatitis B virus causing hepatoma). As for heredity, in the majority of cases it acts probably by increasing the mutation rate, and this may apply to both high penetrance genes and to low penetrance genes; on the other hand, sometimes an oncogene with a germ-line mutation appears to be in lieu of the first somatic mutation, for instance in the case of *RET* in Multiple Endocrine Neoplasia type 2, thus decreasing by one the number of mutations required for the development of cancer (see Figure 9).

In order to understand the pathogenesis of tumors we must consider their very extensive variety: not only can they arise in virtually every possible cell type in the body, but even within the set of tumors arising from a specific type of cell there is marked heterogeneity, some of it well explored and some yet to be unravelled. The somatic mutation-Darwinian selection model of cancer is appropriately versatile: we can presume, and we know in specific cases that different genes are involved: some 400 have been already identified[18]. To this end, the methodology that has given the highest returns has been cytogenetic analysis, which has spotted (i) chromosomal translocations harbouring fusion genes or rearrangements that dysregulate gene expression, as well as (ii) loss of heterozigosity betraying deletions. In other cases somatic mutations have been discovered in genes already known to have germ-line mutations in cancer-prone families, or by deliberately testing for somatic mutations in candidate genes. Not surprisingly, many of the genes involved belong to sets that are relevant to broad functions within the cell (the buzz-term today is *gene ontology*): particularly the cell cycle, signalling, regulation of transcription, apoptosis and, once again, genome stability (DNA repair)[19,20].

**Figure 10:**
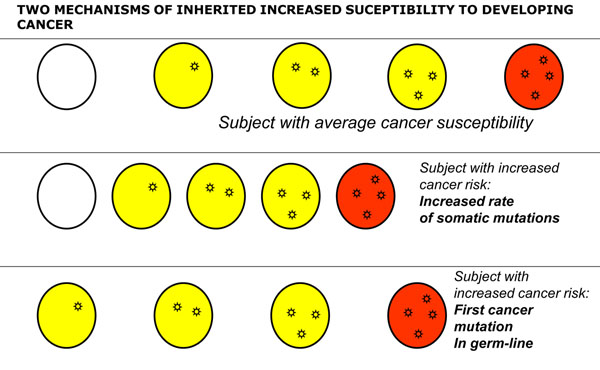
**A graphic representation (current referred to as a cyclo-plot) of the multiple defects detected in the genome of a tumor (a small-cell lung cancer cell line) by deep sequencing**. Individual chromosomes are depicted on the outer circle followed by concentric tracks for point mutation, copy number and rearrangement data relative to mapping position in the genome. Arrows indicate examples of the various types of somatic mutation present in this cancer genome. From Stratton et al., 2009 (ref. 29).

**Figure 11:**
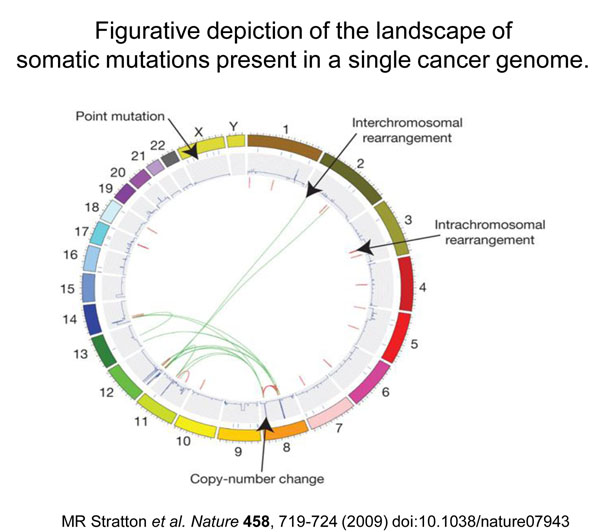
Figurative depiction of the landscape of somatic mutations present in a single cancer genome.

The search for molecular lesions in tumors has taken on a new dimension thanks to two powerful technologies: the micro-arrays for quantitative analysis of global gene expression[21-25] (the *transcriptome*); and ‘deep’ sequencing for the global analysis of the entire genome (or at least the *exome*). The former offers the most complete phenotypic characterization of a tumor we could ever hope for – we could call this the *ultimate phenotype*; the latter can identify all the somatic mutations in an individual tumor – we could call this the *somatic genotype* (see Figure 10). The ground-breaking paper[26] on the latter was published in 2006; and already it has been followed by a flurry of similar work on different types of tumors[27-30]. The somatic genotype of the tumor can be fully characterized by sequencing in parallel (from non-tumor DNA) also the inherited genome of the patient: thus, the issue of inherited variation versus acquired somatic mutation can be rigorously circumvented. A more difficult issue has to do with the fact that somatic mutations can occur (indeed are relatively common) in any normal cell: therefore a somatic mutation found in a tumor does non automatically qualify as being causative of that tumor; therefore we must improve algorithms aiming to disentangle *driver mutations* (*i.e.* pathogenic mutations) from *passenger mutations*. At any rate, by this approach not only are new genes being identified; also, patterns of mutations are emerging (Figure 11) that may be signatures of exposure to individual environmental mutagens [26]: an unexpected bonus of molecular studies that is highly relevant to the focus of this meeting.

**Figure 12:**
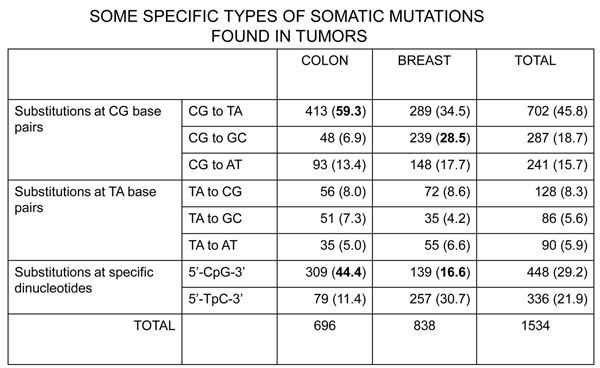
**A cartoon illustrating the central role of chance in cancer formation**, based on the fact that somatic mutations are stochastic events. Inherited factors (see text and Fig. 6) can modulate the process, but they somatic mutations are still needed for the onset of cancer; and environmental factors work in large measure by increasing either the mutation rate (mutagenic agents) or the number of cell divisions (*e.g.* with an inflammatory process, such as one caused for instance by *Helicobacter pylori*).

Somatic mutations are central to the process of oncogenesis, because almost certainly no tumor can arise without them (although epigenetic phenomena are important [31-33], I think it is highly unlikely that gene silencing by promoter methylation alone can do the job). The rate of somatic mutations – and thus the risk of cancer – can be increased by inherited genes or by environmental agents, as we have outlined; however, somatic mutations occur all the time as spontaneous stochastic events, because the replication of DNA is extremely faithful but nor perfect: this means that there is always an element of chance in oncogenesis (Figure 12). In this respect, we know surprisingly little about the normal baseline somatic mutation rate (*μ*). Over the past several years, by using the X-linked gene *PIG-A* as a sentinel gene, we have developed a relatively simple methodology to measure *μ* in any individual[34-36]: we have determined the normal range of *μ*, and we have shown that it is higher in severak groups of cancer-prone subjects. It will be important to determine whether *μ* correlates with the risk of sporadic cancer, and whether we can measure changes in *μ* in subjects who are exposed to environmental carcinogens. It is also not unconceivable that *μ* could be decresed by pharmacological agents.

**Figure 13:**
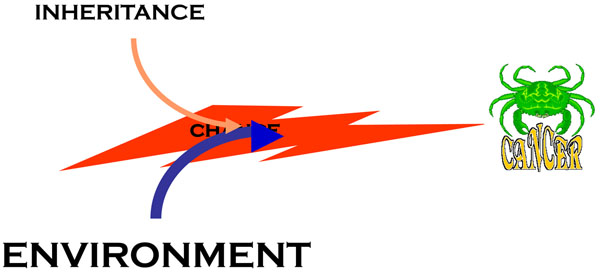
**A new methodology makes it relatively easy to measure in any individual the intrinsic rate of somatic mutation**. In two conditions known to be associated with cancer proneness the rate of somatic mutation is markedly increased over that observed in a control group.

The progress of contemporary biology has led us within thirty years from a multitude of theories about oncogenesis to the established fact that cancer is a genetic disorder of somatic cells. On the other hand, much recent literature gives the impression that there is a surplus of information, from gene expression profiles to proteomics to metabolomics, with the risk that while we are truly ‘drowned by data, we remain thirsty for knowledge’. If we want to heed the teachings of Lorenzo Tomatis, I think the message is clear: we ought to take advantage of the new powerful technologies – not by becoming their slaves, but remaining their masters. Identifying somatic mutations in a tumor is important not because it qualifies for ‘oncogenomics’, but because through a deeper understanding of the nature of that particular tumor it can help us to optimize therapy or to design new therapeutic approaches. Figure 13.

## Competing interests

The author declare that has no competing financial or non-financial interests.
